# A clinical dilemma amid COVID-19 pandemic: missed or encountered diagnosis of cancer?

**DOI:** 10.2217/fon-2020-0501

**Published:** 2020-06-21

**Authors:** Emre Yekedüz, Ayşe Müge Karcıoğlu, Güngör Utkan, Yüksel Ürün

**Affiliations:** ^1^Ankara University Faculty of Medicine Department of Medical Oncology, Ankara, Turkey; ^2^Ankara University Cancer Research Institute, Ankara, Turkey; ^3^Department of Intensive Care, Ministry of Health Ankara City Hospital, Ankara, Turkey

**Keywords:** COVID-19, incidental cancer diagnosis, missed cancer diagnosis

After the first case of severe acute respiratory syndrome-coronavirus 2 (SARS-CoV2) was observed in China in December 2019, coronavirus infection 2019 (COVID-19) was declared as a pandemic by WHO on 11 March 2020 [1]. As of 11 May 2020, the COVID-19 pandemic is still a significant problem worldwide. It has affected the healthcare systems in the majority of European countries and, particularly, the USA. Several nonemergent interventions, including cancer screening and follow-up visits, have been canceled due to the COVID-19 pandemic. Screening is crucial for early diagnosis and prevents advanced-stage cancer, and there are some concerns regarding the disruption of cancer screening during the pandemic. According to IQVIA Institute for Human Data Science, cancer screening decreased by 90% in April 2020 compared with February 2020 in the USA. The rate of colonoscopies, mammograms, pap smears, PSA tests and CT scans decreased by 90, 87, 83, 60 and 39%, respectively. Additionally, the number of admissions to the oncology outpatient clinics decreased by up to 50% [2]. Furthermore, current clinical guidelines are supporting the delaying routine control visits or using telemedicine systems for patients in remission [3]. At this point there is a growing concern regarding the imminent increase in advanced cancer diagnoses. IQVIA Institute for Human Data Science claimed that 80,000 missed diagnoses of cancer will be observed in the USA in the 3 months to early June 2020. Furthermore, the majority of these cases will be breast cancers [2]. Outside the USA, there has an interruption in cancer screening due to COVID-19 in European countries. As of 22 May 2020, 44,700 confirmed cases and 5775 deaths due to COVID-19 were observed in the Netherlands [4]. Furthermore, compared with cancer diagnosis rates in the period before the first COVID-19 diagnosis in the Netherlands, there is a decrease in newly diagnosed skin and non-skin cancers by up to 74 and 40%, respectively [5].

Chest CT has been used widely not only suspected but also confirmed COVID-19 cases. According to the American College of Radiology guideline, it should not be used for COVID-19 screening and diagnosis because they emphasized that chest CT findings of COVID-19 might be confused with the other infectious diseases such as influenza. Furthermore, if the patient does not have a positive viral test result for COVID-19, it might affect the patient’s management and treatment due to false chest CT findings [6]. Despite this recommendation, it is still used in the majority of hospitalized cases to diagnose pneumonia. Indeed, SARS-CoV2 affects the lower respiratory system and it causes lung infections ranging from mild pneumonia to severe lung infections that result in acute respiratory distress syndrome. The chest CT findings, such as bilateral ground-glass opacity, are similar to the other lung infections. Furthermore, there are no particular criteria to distinguish COVID-19 related lung infections from other pneumonia causes [7].

In a study, the number of incidental cancer diagnosis was 4% among cancer patients. Koo *et al.* showed that 1 out of 25 patients having cancer diagnosis was diagnosed by incidentally while performing blood tests or imaging due to other pre-existing conditions [8]. Furthermore, in 2015, Gould *et al.* estimated that 4.8 million people had chest CT scan in the USA and 63,000 of them (1.3% of all chest CT scans) had malignant nodules in the lung parenchyma [9]. In this regard, we have some following questions for cancer diagnosis amid the COVID-19 pandemic: in compliance with the increased number of patients who are performed chest CT scans, does the number of cancer patients who are incidentally diagnosed increase? Despite decrements in the cancer screening through usual ways (e.g., colonoscopy, mammography), can chest CT scan cause an increased incidental diagnosis of cancer? And most importantly, when the SARS-CoV2 test is positive, how should we approach this patient if the patient is suspected of being diagnosed with cancer due to chest CT findings?

We do not have information about the total daily numbers of chest CT scans during the pandemic in the USA and Europe yet. However, the number of chest CT scans are expected to increase during the pandemic. Despite the recommendations of American College of Radiology that chest CT should not be used as a diagnostic tool for COVID-19, it plays a crucial role in determining the severity of the infection in hospitalized patients in particular [10]. Thus, in compliance with the increased number of hospitalized patients, the number of chest CT scans has been increasing during the pandemic. Finally, in parallel with the increased number of chest CT scans, the number of incidental cancer diagnoses are expected to increase. In addition to the findings of COVID-19 pneumonia, pulmonary nodules, enlarged lymph nodes and bone metastases can be observed by incidentally [11]. Fortunately, we have current guidelines regarding the management of patients with suspected malignancy during the pandemic. In this context, a new guideline has been released by the European Society for Medical Oncology. According to this guideline, the patients who have suspicious nodules or mass on the chest CT scans should be performed further diagnostic procedures and biopsies as well [12]. Additionally, a new guideline has also been released by the American College of Chest Physicians. They recommend delaying cancer screening for smaller lung nodules than 8 mm; however, they recommend performing advanced diagnostic procedures for nodules that have a higher risk for cancer [13]. According to the Center for Disease Control and Prevention guideline, diagnostic procedures should be postponed if delaying them has not a harmful result for the individual [14]. However, there is no clear recommendation regarding the patient’s management if the patient has an active COVID-19 infection when a highly suspicious nodule or mass on a chest CT scan is detected. In this case, taking action according to the patient’s clinical condition might be a reasonable approach. For instance, if the patient has highly suspected malignancy findings on a chest CT scan in the intensive care unit, the diagnostic procedures should be delayed until the patient is well. Besides, the severity of the COVID-19 pandemic in the local area should be taken into account for the management of these patients.

There is still a debate regarding decrement in cancer screening and new cancer diagnoses during the pandemic. However, the number of cancer cases diagnosed incidentally may increase and there is no clear advice on how we manage these patients during and most importantly, after COVID-19 infection. We have to take into account these patients and plan concerning the diagnosis and treatment of cancer in these patients because it seems that we might need more evidence-based recommendations for the management of them in the near future.
